# Methylglyoxal attenuates isoproterenol-induced increase in uncoupling protein 1 expression through activation of JNK signaling pathway in beige adipocytes

**DOI:** 10.1016/j.bbrep.2021.101127

**Published:** 2021-09-06

**Authors:** Su-Ping Ng, Wataru Nomura, Haruya Takahashi, Kazuo Inoue, Teruo Kawada, Tsuyoshi Goto

**Affiliations:** a Laboratory of Molecular Function of Food, Division of Food Science and Biotechnology, Graduate School of Agriculture, Kyoto University, Uji, Kyoto 611-0011, Japan; b Research Unit for Physiological Chemistry, The Center for the Promotion of Interdisciplinary Education and Research, Kyoto University, Kyoto 606-8317, Japan

**Keywords:** Methylglyoxal, Beige adipocytes, *Ucp1*, JNK, MG, methylglyoxal, iWAT, inguinal white adipose tissue, PKA, protein kinase A, BBGC, S-p-bromobenzylglutathione cyclopentyl diester, HSL, hormone-sensitive lipase, CREB, cAMP response element-binding protein, NEFA, non-esterified fatty acids, JNK, c-Jun N-terminal kinase, ERK, extracellular receptor kinase, NAC, N-acetyl-l-cysteine, SEM, standard error of the mean

## Abstract

Methylglyoxal (MG) is a metabolite derived from glycolysis whose levels in the blood and tissues of patients with diabetes are higher than those of healthy individuals, suggesting that MG is associated with the development of diabetic complications. However, it remains unknown whether high levels of MG are a cause or consequence of diabetes. Here, we show that MG negatively affects the expression of uncoupling protein 1 (UCP1), which is involved in thermogenesis and the regulation of systemic metabolism. Decreased *Ucp1* expression is associated with obesity and type 2 diabetes. We found that MG attenuated the increase in *Ucp1* expression following treatment with isoproterenol in beige adipocytes. However, MG did not affect protein kinase A signaling, the core coordinator of isoproterenol-induced *Ucp1* expression. Instead, MG activated c-Jun N-terminal kinase (JNK) and p38 mitogen-activated protein kinases. We found that JNK inhibition, but not p38, recovered isoproterenol-stimulated *Ucp1* expression under MG treatment. Altogether, these results suggest an inhibitory role of MG on the thermogenic function of beige adipocytes through the JNK signaling pathway.

## Highlights

• Methylglyoxal suppresses isoproterenol-induced *Ucp1* expression in beige adipocytes.
• Methylglyoxal activates JNK in beige adipocytes.
• Inhibition of JNK recovers methylglyoxal-suppressed *Ucp1* expression.

## 1 Introduction

Adipocytes, which play an important role in lipid metabolism, are generally classified into white and brown adipocytes according to their function [1]. White adipocytes store excess energy in the form of triglycerides (TG), while brown adipocytes produce heat associated with energy consumption through the degradation of TG, which plays a vital role in regulating systemic metabolism and thermogenesis [1]. Uncoupling protein 1 (UCP1), a proton carrier located in the inner membrane of the mitochondria, is involved in the thermogenic function of brown adipocytes [2,3]. UCP1 activation causes dissipation of the electrochemical proton gradient and a decrease in the proton motive force used to synthesize ATP. Proton leakage induced by UCP1 uncouples the proton gradient from ATP synthesis, releasing the free energy as heat [2,3]. Recently, some white adipocytes located in subcutaneous fat, such as the inguinal white adipose tissue (iWAT), have been shown to increase *Ucp1* expression under cold exposure or adrenergic stimulation (i.e., brown-like “beige” adipocytes) [4,5]. Beige adipocytes also expend energy through UCP1-mediated thermogenesis [4,5]. Therefore, the elucidation of the molecular mechanisms underlying the regulation of thermogenesis in both brown and beige adipocytes is of interest because it can provide novel insights into approaches to control and treat obesity and obesity-related metabolic diseases such as type 2 diabetes and diabetic complications [4,5].

Although the development of diabetic complications is the result of a highly complex process, the accumulation of advanced glycation end products (AGEs), the synthesis of which is initiated by a non-enzymatic reaction between the aldehyde groups of glucose and amino groups of proteins, is recognized as one of the major factors linked to it [6]. Methylglyoxal (MG, CH_3_COCHO) is a ubiquitous 2-oxoaldehyde derived from glycolysis [[7], [8], [9]]. Although MG is a natural metabolite, it is highly reactive because it contains two carbonyl groups and has a higher potential than glucose to produce AGEs. Plasma and tissue MG levels are higher in patients with diabetes than in healthy individuals, suggesting that MG plays a role in the development and progression of diabetic complications [[10], [11], [12]]. MG is mainly metabolized to d-lactate within cells through a ubiquitous glutathione-dependent glyoxalase system consisting of glyoxalase I and glyoxalase II [7,9]. Functional genome analyses suggest a relationship between a deficiency in this system, which implies increased MG and the development of diabetic complications [13,14]. However, it remains to be determined whether high MG levels are a cause or consequence of diabetes.

In this study, we investigated the effect of MG on *Ucp1* expression induced by treatment with the β-adrenergic receptor agonist, isoproterenol, in differentiated immortalized iWAT-derived pre-adipocytes. Although the isoproterenol-induced expression of *Ucp1* was inhibited by MG, downregulation of the protein kinase A (PKA) pathway, which contributes to *Ucp1* expression by isoproterenol, was not responsible for this inhibitory effect. We found that MG enhanced the phosphorylation of c-Jun N-terminal kinase (JNK) mitogen-activated protein kinase (MAPK), and an inhibitor of JNK suppressed the inhibitory effect of MG on *Ucp1* expression. These results indicate that activation of JNK MAPK is necessary for the MG-induced inhibition of *Ucp1* expression.

## 2 Materials and methods

### 2.1 Materials

Dulbecco's modified Eagle's medium (DMEM)-high glucose, insulin, indomethacin, SB203580, and N-acetyl-l-cysteine (NAC) were purchased from Wako (Osaka, Japan). Fetal bovine serum was purchased from Gibco (FBS; Grand Island, NY, USA). Penicillin-Streptomycin mixed solution, 3-isobutyl-1-methylxanthine (IBMX), dexamethasone, and dihydroxyacetone (DHA) were purchased from Nacalai Tesque (Kyoto, Japan). Rosiglitazone was purchased from LKT Laboratories (Minneapolis, MN, USA). 3,3′,5′-triiodo-l-thyronine (T_3_), S-p-bromobenzylglutathione cyclopentyl diester (BBGC), MG, and isoproterenol were purchased from Sigma-Aldrich (MO, USA). SP600125 was purchased from Enzo Life Sciences (NY, USA).

### 2.2 Cell culture

Immortalized primary pre-adipocytes from mouse iWAT were a kind gift from Dr. Shingo Kajimura (Harvard Medical School, MA, USA). C3H10T1/2 cells were purchased from the American Type Culture Collection (Manassas, VA, USA). These cells were maintained in a humidified 5% CO_2_ atmosphere at 37 °C in basic medium (DMEM-high glucose supplemented with 10% (v/v) FBS, 10,000 units/ml penicillin, and 10,000 μg/ml streptomycin). To differentiate the pre-adipocytes into mature adipocytes, cells were cultured to confluence before stimulation with 1 nM T_3_, 5 μg/ml insulin, 0.5 μM rosiglitazone, 2 μg/ml dexamethasone, 0.5 mM IBMX, and 125 μM indomethacin in a basic medium for 48 h. The medium was then replaced with a growth medium (basic medium supplemented with 1 nM T_3_, 5 μg/ml insulin, and 0.5 μM rosiglitazone). This step was repeated every 2 days until the adipocytes reached the 6th day after the induction of differentiation. After 6 days of differentiation, the matured adipocytes were incubated in a serum-free medium for 3–5 h before being subjected to 1 μM isoproterenol. The cells were pre-treated with either MG, BBGC or DHA in serum-free medium for 30 min before isoproterenol stimulation. The use of 10 μM SP600125 (JNK inhibitor), 10 μM SB203580 (p38 inhibitor), or 10 mM NAC (reactive oxygen species [ROS] inhibitor) precedes MG or BBGC treatment by 30 min.

### 2.3 RNA preparation and quantification of gene expression

Total RNA was isolated from cultured cells using Sepasol Super-I (Nacalai Tesque, Kyoto, Japan) following the manufacturer's protocol. Total RNA was reverse-transcribed using M-MLV reverse transcriptase (Promega, WI, USA) according to the manufacturer's instructions using a thermal cycler (Takara PCR Thermal Cycler SP, Takara, Shiga, Japan). mRNA expression was quantified by real-time PCR using the SYBR® Green I assay system performed with a LightCycler (Roche Diagnostics, Mannheim, Germany). The protocol for amplification was as follows: denaturation at 95 °C for 15 s, annealing at 60 °C for 15 s, and extension at 72 °C for 45 s. The expression levels of these genes were normalized to those of *36B4*. The primer sequences are listed in Supplementary Table 1.

### 2.4 Luciferase reporter assay

4.5 μg pUCP1-pro-Luc, a tk-LUC luciferase reporter plasmid containing the 3.8-kb portion of the 5′-flanking region of the mouse *Ucp1* gene [15], was transfected into 90% confluent C3H10T1/2 cells growing on a 100-mm culture dish along with 500 ng pGL4.74 (hRluc/TK) vector as an internal control using Lipofectamine 2000 (Thermo Fisher Scientific, MA, USA). Four hours after transfection, the cells were seeded in 96-well-plates overnight. The next day, after 3–5 h of serum starvation, cells were treated with 1 mM MG for 30 min before 1 μM isoproterenol stimulation for 6 h. The cells were lysed and luciferase assay was performed using a Dual-Luciferase Reporter Gene Assay system (Promega, WI, USA) in accordance with the manufacturer's protocol. Luminescence was measured using the Centro XS^3^ LB 960 Microplate Luminometer (Berthold Technologies, Bad Wildbad, Germany).

### 2.5 Western blotting

Western blotting was performed to evaluate protein phosphorylation, as described previously [16]. The antibodies used were anti-phospho-HSL Ser660, #4126; anti-HSL, #4107; anti-phospho-PKA substrate, #9624; anti-perilipin, #9349; anti-phospho-CREB Ser133, #9198; anti-CREB, #9197; anti-β-actin, #4967; anti-phospho-p38 Thr180/Tyr182, #9215; anti-p38, #9212; anti-phospho-JNK Thr183/Tyr185, #9251; anti-JNK, #9252; anti-phospho-ERK Thr202/Tyr204, #9101; and anti-ERK, #9102. All primary antibodies were purchased from Cell Signaling Technology (Danvers, MA, USA). Immunoreactive bands were detected using anti-rabbit HRP secondary antibody, NBP1-75297 (Novus Biologicals, CO, USA) with Immobilon Western Chemiluminescent Horseradish Peroxidase Substrate (Millipore, Burlington, MA, USA) and an LAS-4000 mini-imaging system (Fujifilm, Tokyo, Japan).

### 2.6 Lipolysis assay

Mature adipocytes (day 6 post differentiation induction) were starved of serum for 3–5 h before adrenergic stimulation with 1 μM isoproterenol in serum-free medium containing 2% bovine serum albumin (Nacalai Tesque, Kyoto, Japan) for 3 h. Non-esterified fatty acids (NEFA) and glycerol levels within the cell culture medium were measured using NEFA C (Wako, Osaka, Japan) and triglyceride E (Wako, Osaka, Japan) assay kits, respectively. NEFA and glycerol levels were then normalized to the cellular protein levels.

### 2.7 Intracellular ROS assay

The intracellular levels of ROS were detected using the fluorescent probe H_2_DCFDA (Thermo Fisher Scientific, MA, USA). The adipocytes were incubated with 2 μM of the dye in either the presence or absence of 10 mM NAC for 30 min at 37 °C in the dark, followed by treatment with either 500 μM H_2_O_2_ (Wako, Osaka, Japan) or 1 mM MG for 3 h. Fluorescence levels were then measured in the fluorescence reader, Tecan Infinite F-200 microplate reader (Tecan Inc., Maennedorf, Switzerland) with excitation/emission at 485 nm/535 nm.

### 2.8 Statistical analysis

Statistical analyses were conducted using GraphPad Prism (version 9.2; San Diego, CA, USA). After confirming that datasets fulfill the Shapiro-Wilk normality test, statistical significance was determined using one-way ANOVA followed by Sidak's or Dunnett's multiple comparison test, as indicated in the figure legends. Differences were considered significant at P < 0.05.

## 3 Results and discussion

### 3.1 MG attenuates the isoproterenol-induced expression of *Ucp1*

**Fig. 1:**
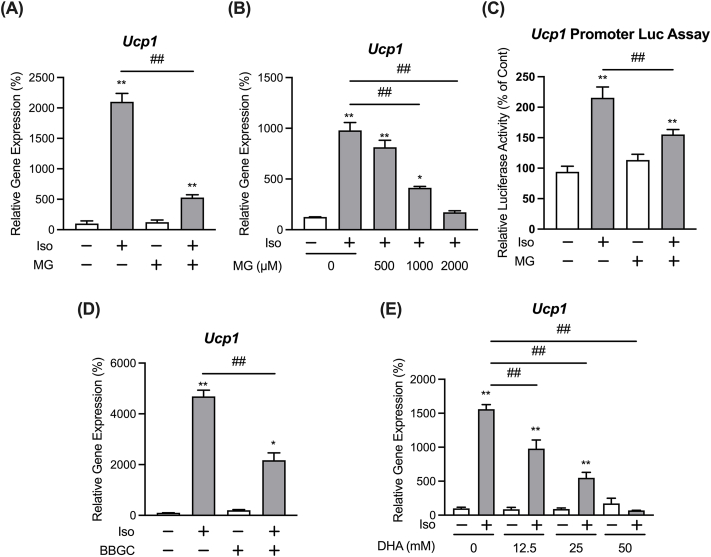
MG represses isoproterenol-stimulated *Ucp1* expression in differentiated immortalized iWAT-derived pre-adipocytes. Adipocytes were treated with 1 mM MG (A), or 0.5, 1, or 2 mM MG (B) for 30 min, followed by stimulation with 1 μM isoproterenol (Iso) for 3 h. mRNA expression levels of *Ucp1* in the cells were then determined by real-time PCR. Data are presented as the mean ± SEM (error bars), with n = 3–4 per group. (C) C3H10T1/2 fibroblasts were transfected with the *Ucp1* promoter reporter plasmid, pUCP1-pro-Luc. After that, the cells were treated with 1 mM MG for 30 min, followed by stimulation with 1 μM isoproterenol for 6 h. Cells were lysed and luciferase assay was then performed. Data are presented as the mean ± SEM (error bars), with n = 5 per group. 20 μM BBGC (D), or 12.5, 25 or 50 mM DHA (E) were pre-treated in adipocytes for 30 min, followed by stimulation with 1 μM isoproterenol for 3 h. Then, mRNA expression levels of *Ucp1* in the cells were determined by real-time PCR. Data are presented as the mean ± SEM (error bars), with n = 3–4 per group. One-way ANOVA followed by Dunnett's (A,D) or Sidak's (B,C,E) post-hoc tests were done to determine statistical significance. *, P < 0.05; **, P < 0.01 between the non-stimulated vehicular control group and the respective groups. #, P < 0.05; ##, P < 0.01.

To examine whether MG affects *Ucp1* expression, we determined *Ucp1* expression following stimulation by a non-selective β-adrenergic receptor agonist, isoproterenol, in differentiated immortalized iWAT-derived pre-adipocytes. As shown in Fig. 1A, pretreatment with MG decreased *Ucp1* expression induced by 3 h of isoproterenol treatment. The inhibitory effect of MG on the isoproterenol-induced expression of *Ucp1* was dose-dependent (Fig. 1B). In addition, usage of a luciferase reporter system show that MG significantly decreased the luciferase activity of the mouse *Ucp1* gene promoter reporter plasmid under isoproterenol stimulation, which suggests that MG attenuates the isoproterenol-induced expression of *Ucp1* through decreasing its promoter activity (Fig. 1C).

Glyoxalase I is the main metabolic enzyme for MG; therefore, treatment with BBGC, an inhibitor of glyoxalase I, increases the concentration of intracellular MG [17]. Moreover, BBGC pretreatment negatively affected the isoproterenol-induced expression of *Ucp1* (Fig. 1D). DHA is the smallest ketotriose that is utilized by many organisms as an energy source. It has been previously reported that DHA is non-enzymatically converted to MG [18], and intracellular MG levels in yeast are increased by cultivation under presence of DHA [19]. The isoproterenol-induced expression of *Ucp1* was attenuated by treatment with DHA (Fig. 1E). These results suggest that intracellular MG negatively influences the expression of *Ucp1* induced by adrenergic stimulation. Next, we set out to examine the mechanism by which intracellular MG affects *Ucp1* expression.

### 3.2 MG does not affect the isoproterenol-induced activation of PKA signaling pathway

**Fig. 2:**
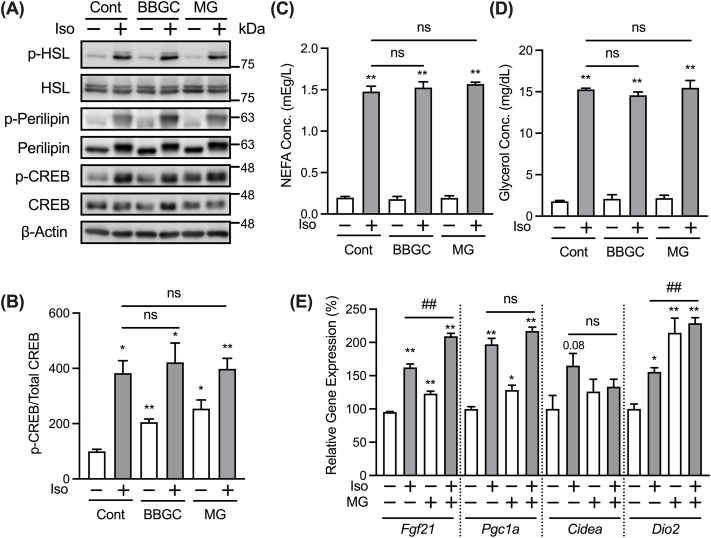
MG does not suppress the isoproterenol-stimulated PKA lipolytic pathway. (A) Adipocytes were treated with 20 μM BBGC or 1 mM MG for 30 min before stimulation with 1 μM isoproterenol (Iso) for 30 min. Phosphorylation of HSL (p-HSL), protein levels of HSL, phosphorylation of perilipin (p-perilipin), protein levels of perilipin, phosphorylation of CREB (p-CREB), protein levels of CREB, and protein levels of β-actin were determined using anti-phospho HSL Ser660, anti-HSL, anti-phospho-PKA substrate, anti-perilipin, anti-phospho CREB Ser133, anti-CREB, and anti-β-actin antibodies respectively. (B) Phosphorylation levels of CREB were quantified by measuring the intensity of the immunoreactive bands using ImageJ. The ratio of p-CREB/CREB in the non-stimulated vehicular control group was assigned a relative value of 100. Data are presented as the mean ± SEM (error bars), with n = 5 per group. (C,D) Adipocytes were treated with 20 μM BBGC or 1 mM MG for 30 min before stimulation with 1 μM isoproterenol (Iso) for 3 h. NEFA (C) and glycerol (D) levels in the culture medium were measured and normalized to cellular protein levels. Data are presented as the mean ± SEM (error bars), with n = 3–4 per group. (E) Expression levels of thermogenic genes in cells treated with 1 mM MG for 30 min before stimulation with 1 μM isoproterenol (Iso) for 3 h. Data are presented as mean ± SEM (error bars), with n = 3–4 per group. One-way ANOVA followed by Dunnett's (B,D) or Sidak's (C,E) post-hoc tests were done to determine statistical significance. *, P < 0.05; **, P < 0.01 between the non-stimulated vehicular control group and the respective groups. #, P < 0.05; ##, P < 0.01.

Stimulation of β-adrenergic receptors on adipocytes by adrenergic agonists increases the intracellular level of cAMP, which in turn activates PKA [20]. Activated PKA then directly phosphorylates its target proteins, including hormone-sensitive lipase (HSL) and perilipin (a protein located on the lipid droplet surface), which are crucial for the activation of lipolysis [21,22]. The PKA signaling pathway also contributes to the increased expression of *Ucp1* induced by adrenergic stimulation via activation of cAMP response element-binding protein (CREB) in adipocytes [23]. Since MG inhibited *Ucp1* expression induced by isoproterenol, we next examined the phosphorylation levels of HSL, perilipin, and CREB as indicators of PKA activity to determine if MG negatively affected the activation of PKA signaling. Ser660 at HSL and Ser133 at CREB are known as the target sites of PKA [21,24], and perilipin has multiple PKA consensus sequences, the phosphorylation levels of which can be detected using an anti-phospho-PKA substrate antibody [16,25]. As shown in Fig. 2A and B, the isoproterenol-stimulated increase in the phosphorylation levels of HSL, perilipin, and CREB was not significantly decreased by pretreatment with MG or BBGC. Activation of PKA signaling by adrenergic agonists also enhances adipocyte lipolysis and sequentially increases the release of NEFA and glycerol into the medium [26]. The amounts of NEFA and glycerol released after isoproterenol stimulation were not decreased by MG or BBGC pretreatment (Fig. 2C and D). These results suggest that the MG-induced inhibition of *Ucp1* expression is not due to the attenuated activation of PKA signaling by isoproterenol stimulation. In contrast, treatment with MG or BBGC increased basal levels of CREB phosphorylation (Fig. 2B). Ser133 in CREB is known as a phosphorylation target site of mitogen- and stress-activated protein kinase (MSK1) in addition to PKA [27]. Since MG did not activate PKA signaling, MG might have enhanced CREB phosphorylation through the activation of MSK1.

Isoproterenol stimulation not only increases the expression of *Ucp1*, but also genes related to beige adipocyte function (*Fgf21*, *Pgc1α*, *Cidea*, and *Dio2*) by activating PKA signaling [[28], [29], [30], [31]]. As shown in Fig. 2E, MG did not significantly decrease the isoproterenol-induced expression of these genes. This observation is consistent with the analysis of PKA signaling activity by western blotting, suggesting that MG may specifically inhibit *Ucp1* expression without affecting PKA signaling in response to isoproterenol.

### 3.3 MG attenuates *Ucp1* expression through the activation of JNK signaling pathway

**Fig. 3:**
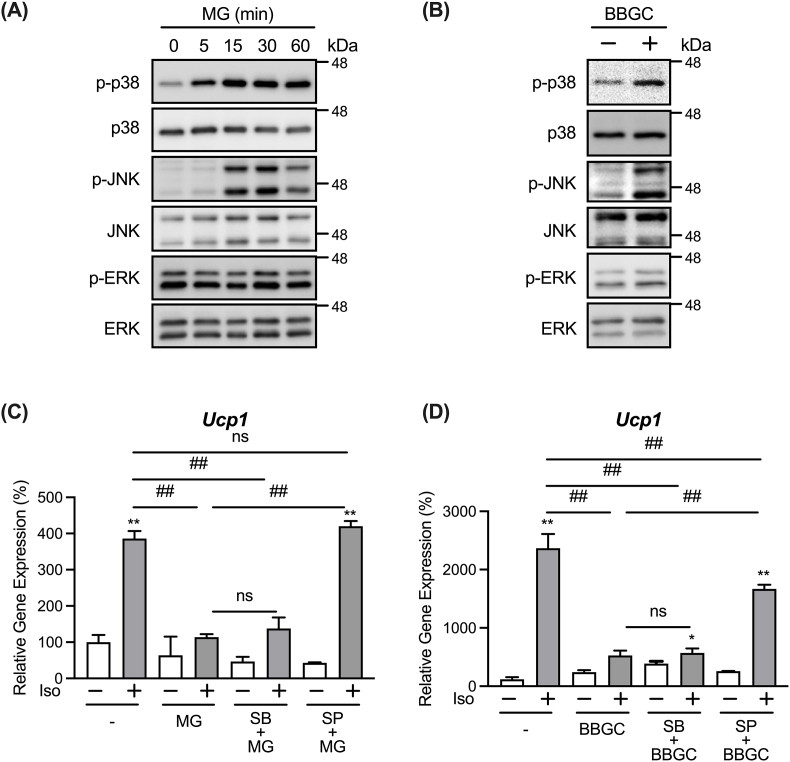
MG represses isoproterenol-induced *Ucp1* expression in a JNK-dependent manner. Adipocytes were treated with 1 mM MG for 5, 15, 30, or 60 min (A) or 20 μM BBGC for 30 min (B). Phosphorylation of p38 (p-p38), protein levels of p38, phosphorylation of JNK (p-JNK), protein levels of JNK, phosphorylation of ERK (p-ERK), and protein levels of ERK were determined using anti-phospho p38 Thr180/Tyr182, anti-p38, anti-phospho JNK Thr183/Tyr185, anti-JNK, anti-phospho ERK Thr202/Tyr204, and anti-ERK antibodies, respectively. (C,D) Adipocytes were pre-treated with 10 μM SB203580 (SB), a p38 inhibitor, or 10 μM SP600125 (SP), a JNK inhibitor, for 30 min. The cells were then treated with either 1 mM MG (C) or 20 μM BBGC (D) for an additional 30 min before stimulation with 1 μM isoproterenol (Iso) for 3 h. mRNA expression levels of *Ucp1* in the cells were determined by real-time PCR. Data are presented as the mean ± SEM (error bars), with n = 3–4 per group. One-way ANOVA followed by Sidak's post-hoc test was done to determine statistical significance for (C,D). *, P < 0.05; **, P < 0.01 between the non-stimulated vehicular control group and the respective groups. #, P < 0.05; ##, P < 0.01.

MG is involved in the activation of signaling pathways as a signaling molecule in diverse organisms from yeast to mammalian cells [7,9]. Moreover, MG induces the activation of JNK, p38, and extracellular receptor kinase (ERK) MAPK signaling pathways in cultured cells [[32], [33], [34]]. As shown in Fig. 3A, MG enhanced the phosphorylation of JNK and p38 MAPKs in adipocytes. An increase in the phosphorylation levels of JNK and p38 was also observed following treatment with BBGC (Fig. 3B). In contrast, ERK phosphorylation levels were not significantly affected by MG or BBGC (Fig. 3A and B).

To determine whether the activation of JNK and p38 participates in the negative effect of MG on *Ucp1* expression, we examined the effect of MAPK inhibitors on the MG-induced inhibition of *Ucp1* expression. Although the p38 MAPK inhibitor, SB203580, did not affect the decrease in the isoproterenol-induced expression of *Ucp1* by MG or BBGC treatment, the JNK MAPK inhibitor, SP600125, recovered this decrease (Fig. 3C and D). These results suggest that MG attenuates the isoproterenol-induced expression of *Ucp1* through the activation of the JNK signaling pathway.

Peroxisome proliferator-activated receptor γ (PPARγ), a member of the nuclear receptor family of ligand-activated transcription factors, is the master regulator of adipogenesis [35]. In addition, PPARγ controls the transcriptional regulation of *Ucp1* [35]. ERK and JNK MAPK phosphorylates PPARγ at Ser112, which attenuates PPARγ activity [36], and this phosphorylation is also associated with *Ucp1* expression [37]. Although these findings raise the possibility that MG inhibits *Ucp1* expression via the negative regulation of PPARγ by increasing its phosphorylation, we found no increase in Ser112 phosphorylation of PPARγ following treatment with MG (data not shown).

The number of mitochondria and UCP1 protein in beige adipocytes strongly depends on the activity of mitophagy [38,39], which is repressed in response to thermogenic activation [40,41]. Meanwhile, MG was recently shown to increase mitophagy in brain endothelial cells [42], and the involvement of JNK as an upstream regulator of mitophagy has been reported [43,44]. Hence, one of the possible molecular mechanisms underlying the MG-induced inhibition of *Ucp1* expression through the activation of the JNK signaling pathway may involve altered mitophagy activity. However, further investigations are necessary.

### 3.4 Effect of ROS generation on the MG-induced inhibition of *Ucp1* expression

**Fig. 4:**
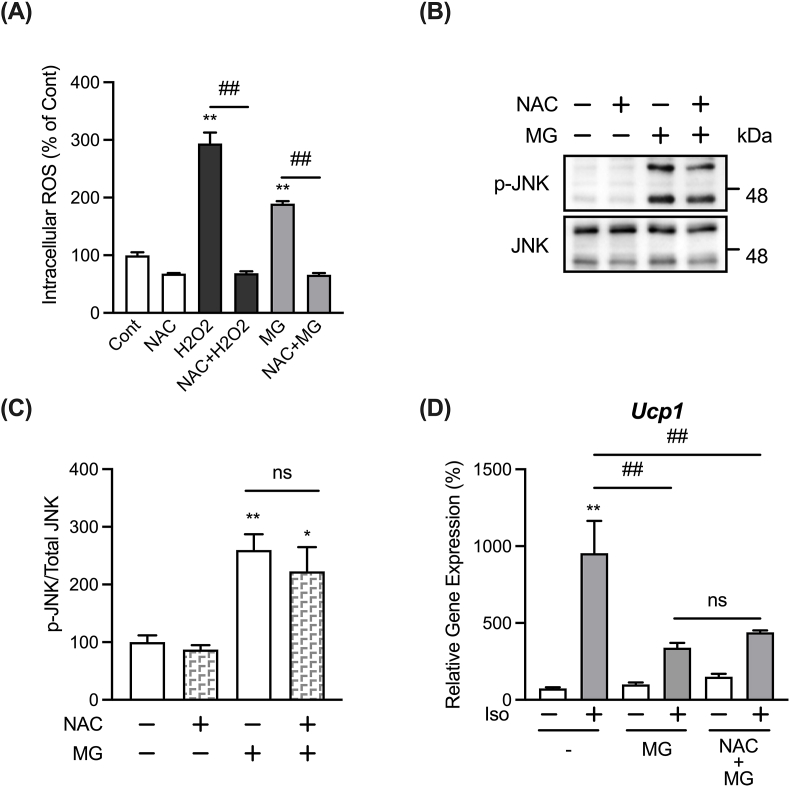
Effect of NAC on MG-induced activation of JNK signaling. (A) Adipocytes were pre-treated with 2 μM H_2_DCFDA with or without 10 mM NAC for 30 min, followed by treatment with either 500 μM H_2_O_2_ or 1 mM MG for 3 h. Intracellular ROS assay was then performed. Data are presented as the mean ± SEM (error bars), with n = 4 per group. (B) Adipocytes were pre-treated with 10 mM NAC, a ROS inhibitor, for 30 min before treatment with 1 mM MG for 1 h. Phosphorylation of JNK (p-JNK) and protein levels of JNK were determined as described in Fig. 3 (A,B). (C) Phosphorylation levels of JNK were quantified by measuring the intensity of the immunoreactive bands using ImageJ. The ratio of p-JNK/JNK in the non-stimulated vehicular control group was assigned a relative value of 100. Data are presented as the mean ± SEM (error bars), with n = 6 per group. (D) After pretreatment of cells with 10 mM NAC and 1 mM MG for 30 min each, cells were stimulated with 1 μM isoproterenol (Iso) for 3 h, and mRNA expression levels of *Ucp1* were determined by real-time PCR. Data are presented as the mean ± SEM (error bars), with n = 3–4 per group. One-way ANOVA followed by Sidak's post-hoc test was done to determine statistical significance for (A,C,D). *, P < 0.05; **, P < 0.01 between the non-stimulated vehicular control group and the respective groups. #, P < 0.05; ##, P < 0.01.

An increase in ROS levels can adversely affect cell function and homeostasis, leading to oxidative stress [45]. In some cell lines, such as human umbilical vascular endothelial cells and pancreatic β-cells, MG has been reported to activate the JNK signaling pathway through ROS generation [46,47]. Therefore, we examined whether the MG-induced phosphorylation of JNK is due to ROS generation in differentiated immortalized iWAT-derived pre-adipocytes using the ROS inhibitor, NAC. Similar to other cell lines, 3 h treatment with MG also generates ROS, which could be inhibited by NAC pretreatment, in the adipocytes (Fig. 4A). However, as shown in Fig. 4B and C, the increase in JNK phosphorylation following treatment with MG was not significantly affected by NAC. Meanwhile, the MG-induced inhibition of *Ucp1* expression also did not significantly recover in the presence of NAC (Fig. 4D). These results suggest that ROS generation is not involved in the MG-induced phosphorylation of JNK. At this stage, the molecular machinery by which MG activates the JNK signaling pathway independent of ROS generation in differentiated immortalized iWAT-derived pre-adipocytes is unknown, but if this can be clarified, it will provide new insights into the regulation of *Ucp1* expression.

Thermogenesis in adipose tissue is related to metabolic diseases such as obesity and type 2 diabetes mellitus [48]. Thermogenesis mediated by UCP1 is an important component of total energy expenditure and contributes to the overall energy balance [48]. Based on our findings, the analysis of MG on the thermogenic function in adipose tissues regarding the inhibitory effect of MG on *Ucp1* expression may lead to further understanding of the relationship between MG and diabetes mellitus. However, since this study especially focused on the regulatory mechanisms of *Ucp1* mRNA expression, further investigations into MG's effect on UCP1 protein levels as well as its thermogenic functionality are needed to understand the physiological functions on adipocytes.

## Declaration of competing interest

The authors declare that they have no known competing financial interests or personal relationships that could have influenced the work reported in this paper.

## References

[1] Saely C.H., Geiger K., Drexel H. (2012). Brown versus white adipose tissue: a mini-review. Gerontology.

[2] Cannon B., Nedergaard J.A.N. (2004). Brown adipose tissue: function and physiological significance. Physiol. Rev..

[3] Wang G., Meyer J.G., Cai W., Softic S., Li M.E., Verdin E., Newgard C., Schilling B., Kahn C.R. (2019). Regulation of UCP1 and mitochondrial metabolism in brown adipose tissue by reversible succinylation. Mol. Cell..

[4] Sidossis L., Kajimura S. (2015). Brown and beige fat in humans: thermogenic adipocytes that control energy and glucose homeostasis. J. Clin. Invest..

[5] Vitali A., Murano I., Zingaretti M.C., Frontini A., Ricquier D., Cinti S. (2012). The adipose organ of obesity-prone C57BL/6J mice is composed of mixed white and brown adipocytes. J. Lipid Res..

[6] Ahmed N. (2005). Advanced glycation endproducts—role in pathology of diabetic complications. Diabetes Res. Clin. Pract..

[7] Inoue Y., Maeta K., Nomura W. (2011). Glyoxalase system in yeasts: structure, function, and physiology. Semin. Cell Dev. Biol..

[8] Allaman I., Bélanger M., Magistretti P.J. (2015). Methylglyoxal, the dark side of glycolysis. Front. Neurosci..

[9] Zemva J., Pfaff D., Groener J.B., Fleming T., Herzig S., Teleman A., Nawroth P.P., Tyedmers J. (2019). Effects of the reactive metabolite methylglyoxal on cellular signalling, insulin action and metabolism - what we know in mammals and what we can learn from yeast. Exp. Clin. Endocrinol. Diabetes.

[10] McLellan A.C., Thornalley P.J., Benn J., Sonksen P.H. (1994). Glyoxalase system in clinical diabetes mellitus and correlation with diabetic complications. Clin. Sci..

[11] Beisswenger P.J., Howell S.K., Touchette A.D., Lal S., Szwergold B.S. (1999). Metformin reduces systemic methylglyoxal levels in type 2 diabetes. Diabetes.

[12] Lapolla A., Flamini R., Vedova A.D., Senesi A., Reitano R., Fedele D., Basso E., Seraglia R., Traldi P. (2003). Glyoxal and methylglyoxal levels in diabetic patients: quantitative determination by a new GC/MS method. Clin. Chem. Lab. Med..

[13] Giacco F., Du X., D'Agati V.D., Milne R., Sui G., Geoffrion M., Brownlee M. (2014). Knockdown of glyoxalase 1 mimics diabetic nephropathy in nondiabetic mice. Diabetes.

[14] Moraru A., Wiederstein J., Pfaff D., Fleming T., Miller A.K., Nawroth P., Teleman A.A. (2018). Elevated levels of the reactive metabolite methylglyoxal recapitulate progression of type 2 diabetes. Cell Metabol..

[15] Sakamoto T., Takahashi N., Sawaragi Y., Naknukool S., Yu R., Goto T., Kawada T. (2013). Inflammation induced by RAW macrophages suppresses UCP1 mRNA induction via ERK activation in 10T1/2 adipocytes. Am. J. Physiol. Cell Physiol..

[16] Ng S.P., Nomura W., Mohri S., Takahashi H., Jheng H.F., Ara T., Nagai H., Ito T., Kawada T., Goto T. (2019). Soy hydrolysate enhances the isoproterenol-stimulated lipolytic pathway through an increase in β-adrenergic receptor expression in adipocytes. Biosci. Biotechnol. Biochem..

[17] Thornalley P.J., Edwards L.G., Kang Y., Wyatt C., Davies N., Ladan M.J., Double J. (1996). Antitumour activity of S-p-bromobenzylglutathione cyclopentyl diester in vitro and in vivo: inhibition of glyoxalase I and induction of apoptosis. Biochem. Pharmacol..

[18] Needham J., Lehmann H. (1937). Intermediary carbohydrate metabolism in embryonic life: glyceraldehyde and glucolysis. Biochem. J..

[19] Nomura W., Aoki M., Inoue Y. (2018). Toxicity of dihydroxyacetone is exerted through the formation of methylglyoxal in Saccharomyces cerevisiae: effects on actin polarity and nuclear division. Biochem. J..

[20] Carmen G.Y., Víctor S.M. (2006). Signalling mechanisms regulating lipolysis. Cell. Signal..

[21] Holm C. (2003). Molecular mechanisms regulating hormone-sensitive lipase and lipolysis. Biochem. Soc. Trans..

[22] Miyoshi H., Souza S.C., Zhang H.H., Strissel K.J., Christoffolete M.A., Kovsan J., Rudich A., Kraemer F.B., Bianco A.C., Obin M.S., Greenberg A.S. (2006). Perilipin promotes hormone-sensitive lipase-mediated adipocyte lipolysis via phosphorylation-dependent and -independent mechanisms. J. Biol. Chem..

[23] Nedergaard J., Golozoubova V., Matthias A., Asadi A., Jacobsson A., Cannon B. (2001). UCP1: the only protein able to mediate adaptive non-shivering thermogenesis and metabolic inefficiency. Biochim. Biophys. Acta.

[24] Gonzalez G.A., Montminy M.R. (1989). Cyclic AMP stimulates somatostatin gene transcription by phosphorylation of CREB at serine 133. Cell.

[25] Choi S.M., Tucker D.F., Gross D.N., Easton R.M., DiPilato L.M., Dean A.S., Monks B.R., Birnbaum M.J. (2010). Insulin regulates adipocyte lipolysis via an Akt-independent signaling pathway. Mol. Cell Biol..

[26] Anthonsen M.W., Rönnstrand L., Wernstedt C., Degerman E., Holm C. (1998). Identification of novel phosphorylation sites in hormone-sensitive lipase that are phosphorylated in response to isoproterenol and govern activation properties *in vitro*. J. Biol. Chem..

[27] Deak M., Clifton A.D., Lucocq L.M., Alessi D.R. (1998). Mitogen- and stress-activated protein kinase-1 (MSK1) is directly activated by MAPK and SAPK2/p38, and may mediate activation of CREB. EMBO J..

[28] Collins S., Yehuda-Shnaidman E., Wang H. (2010). Positive and negative control of Ucp1 gene transcription and the role of β-adrenergic signaling networks. Int. J. Obes..

[29] Villarroya F., Peyrou M., Giralt M. (2017). Transcriptional regulation of the uncoupling protein-1 gene. Biochimie.

[30] Iwase M., Sakai S., Seno S., Yeh Y.S., Kuo T., Takahashi H., Nomura W., Jheng H.F., Horton P., Osato N., Matsuda H., Inoue K., Kawada T., Goto T. (2020). Long non-coding RNA 2310069B03Rik functions as a suppressor of Ucp1 expression under prolonged cold exposure in murine beige adipocytes. Biosci. Biotechnol. Biochem..

[31] Iwase M., Tokiwa S., Seno S., Mukai T., Yeh Y.S., Takahashi H., Nomura W., Jheng H.F., Matsumura S., Kusudo T., Osato N., Matsuda H., Inoue K., Kawada T., Goto T. (2020). Glycerol kinase stimulates uncoupling protein 1 expression by regulating fatty acid metabolism in beige adipocytes. J. Biol. Chem..

[32] Akhand A.A., Hossain K., Mitsui H., Kato M., Miyata T., Inagi R., Du J., Takeda K., Kawamoto Y., Suzuki H., Kurokawa K., Nakashima I. (2001). Glyoxal and methylglyoxal trigger distinct signals for map family kinases and caspase activation in human endothelial cells. Free Radic. Biol. Med..

[33] Liu B.F., Miyata S., Hirota Y., Higo S., Miyazaki H., Fukunaga M., Hamada Y., Ueyama S., Muramoto O., Uriuhara A., Kasuga M. (2003). Methylglyoxal induces apoptosis through activation of p38 mitogen-activated protein kinase in rat mesangial cells. Kidney Int..

[34] Chan W.H., Wu H.J., Shiao N.H. (2007). Apoptotic signaling in methylglyoxal-treated human osteoblasts involves oxidative stress, c-Jun N-terminal kinase, caspase-3, and p21-activated kinase 2. J. Cell. Biochem..

[35] Ma X., Wang D., Zhao W., Xu L. (2018). Deciphering the roles of PPARγ in adipocytes via dynamic change of transcription complex. Front. Endocrinol..

[36] Burns K.A., Heuvel J.P.V. (2007). Modulation of PPAR activity via phosphorylation. Biochim. Biophys. Acta.

[37] Grimaldi B., Bellet M.M., Katada S., Astarita G., Hirayama J., Amin R.H., Granneman J.G., Piomelli D., Leff T., Sassone-Corsi P. (2010). PER2 controls lipid metabolism by direct regulation of PPARγ. Cell Metabol..

[38] Lu X., Altshuler-Keylin S., Wang Q., Chen Y., Sponton C.H., Ikeda K., Maretich P., Yoneshiro T., Kajimura S. (2018). Mitophagy controls beige adipocyte maintenance through a Parkin-dependent and UCP1-independent mechanism. Sci. Signal..

[39] Altshuler-Keylin S., Shinoda K., Hasegawa Y., Ikeda K., Hong H., Kang Q., Yang Y., Perera R.M., Debnath J., Kajimura S. (2016). Beige adipocyte maintenance is regulated by autophagy-induced mitochondrial clearance. Cell Metabol..

[40] Taylor D., Gottlieb R.A. (2017). Parkin‐mediated mitophagy is downregulated in browning of white adipose tissue. Obesity.

[41] Szatmári-Tóth M., Shaw A., Csomós I., Mocsár G., Fischer-Posovszky P., Wabitsch M., Balajthy Z., Lányi C., Győry F., Kristóf E., Fésüs L. (2020). Thermogenic activation downregulates high mitophagy rate in human masked and mature beige adipocytes. Int. J. Mol. Sci..

[42] Kim D., Kim K.A., Kim J.H., Kim E.H., Bae O.N. (2020). Methylglyoxal-induced dysfunction in brain endothelial cells via the suppression of akt/HIF-1α pathway and activation of mitophagy associated with increased reactive oxygen species. Antioxidants.

[43] Kim J.H., Kim H.Y., Lee Y.K., Yoon Y.S., Xu W.G., Yoon J.K., Choi S.E., Ko Y.G., Kim M.J., Lee S.J., Wang H.J., Yoon G. (2011). Involvement of mitophagy in oncogenic K-Ras-induced transformation: overcoming a cellular energy deficit from glucose deficiency. Autophagy.

[44] Park J.H., Ko J., Park Y.S., Park J., Hwang J., Koh H.C. (2017). Clearance of damaged mitochondria through PINK1 stabilization by JNK and ERK MAPK signaling in chlorpyrifos-treated neuroblastoma cells. Mol. Neurobiol..

[45] Newsholme P., Cruzat V.F., Keane K.N., Carlessi R., de Bittencourt, PIH (2016). Molecular mechanisms of ROS production and oxidative stress in diabetes. Biochem. J..

[46] Figarola J.L., Singhal J., Rahbar S., Awasthi S., Singhal S.S. (2014). LR-90 prevents methylglyoxal-induced oxidative stress and apoptosis in human endothelial cells. Apoptosis.

[47] Liu C., Huang Y., Zhang Y., Chen X., Kong X., Dong Y. (2017). Intracellular methylglyoxal induces oxidative damage to pancreatic beta cell line INS-1 cell through Ire1α-JNK and mitochondrial apoptotic pathway. Free Radic. Res..

[48] Jia J.J., Tian Y.B., Cao Z.H., Tao L.L., Zhang X., Gao S.Z., Ge C.R., Lin Q.Y., Jois M. (2010). The polymorphisms of UCP1 genes associated with fat metabolism, obesity and diabetes. Mol. Biol. Rep..

