# Supplementary material for: Methylglyoxal attenuates isoproterenol-induced increase in uncoupling protein 1 expression through activation of JNK signaling pathway in beige adipocytes
Source: Biochem Biophys Rep. 2021 Sep 6;28:101127. doi: 10.1016/j.bbrep.2021.101127 (PMC8430270; doi:10.1016/j.bbrep.2021.101127)
Supplement: Multimedia component 1 [file mmc1.pdf]

Supplementary Table 1. Oligonucleotide primers used for mRNA analysis.

**Gene**	**Forward Primer** (5'→3')	**Reverse Primer** (5'→3')
Ucp1	CAAAGTCCGCCTTCAGATCC	AGCCGGCTGAGATCTTGTTT
36b4	TCCTTCTTCCAGGCTTTGGG	GACACCCTCCAGAAAGCGAG
Fgf21	CACCGCAGTCCAGAAAGTCT	ATCCTGGTTTGGGGAGTCCT
Pgc1α	CCCTGCCATTGTTAAGACC	TGCTGCTGTTCCTGTTTTC
Cidea	ATCACAACTGGCCTGGTTACG	TACTACCCGGTGTCCATTTCT
Dio2	AGCCCATGTAACCAGCACCGGA	CAGTCGCACTGGCTCAGGAC

Raw Data of Fig. 2(a)

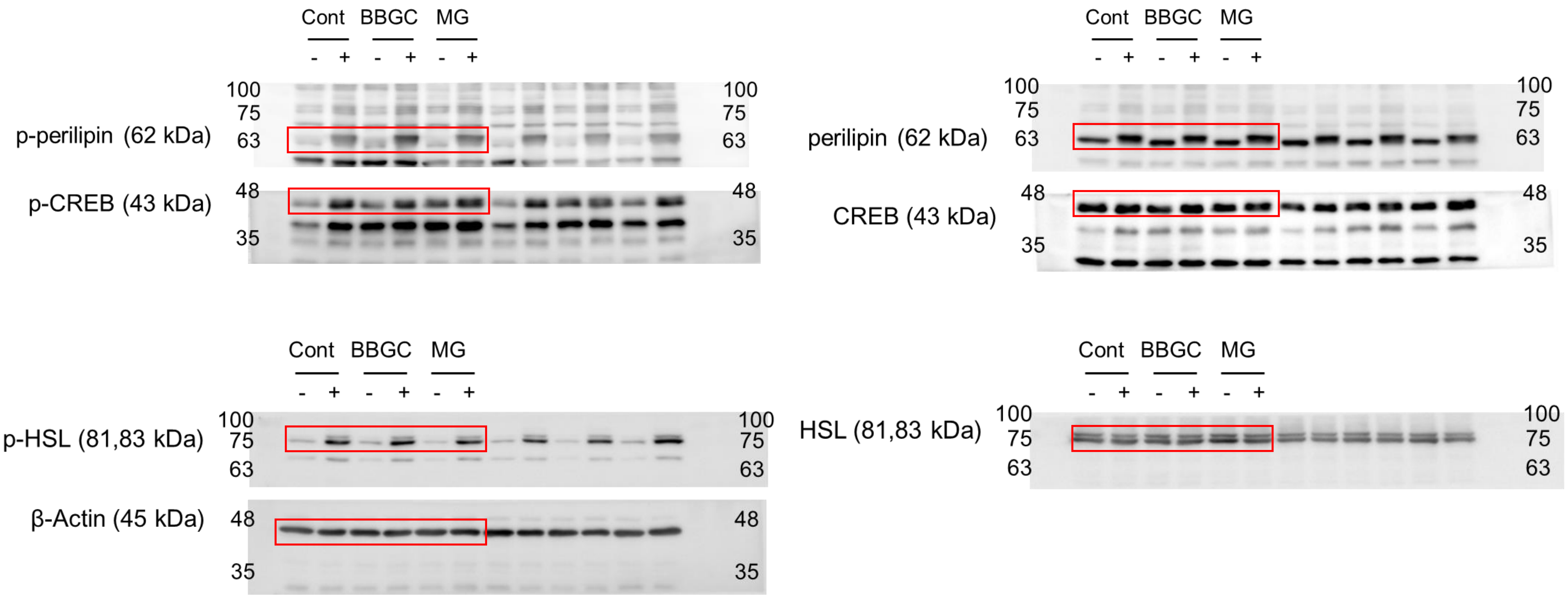

The whole blots of Fig. 2(a) after cutting membranes at molecular weight 48~63 kDa for p-perilipin (62 kDa) and p-CREB (43 kDa) (upper left), or p-HSL (81, 83 kDa) and β-actin (45kDa) (bottom left). Images on the right show membranes that were stripped of their first antibody and re-probed with antibodies for their respective unphosphorylated proteins. The red box indicates the cropped part of the membrane used in Fig 2(a).

Quintuplicates for Fig. 2(b)

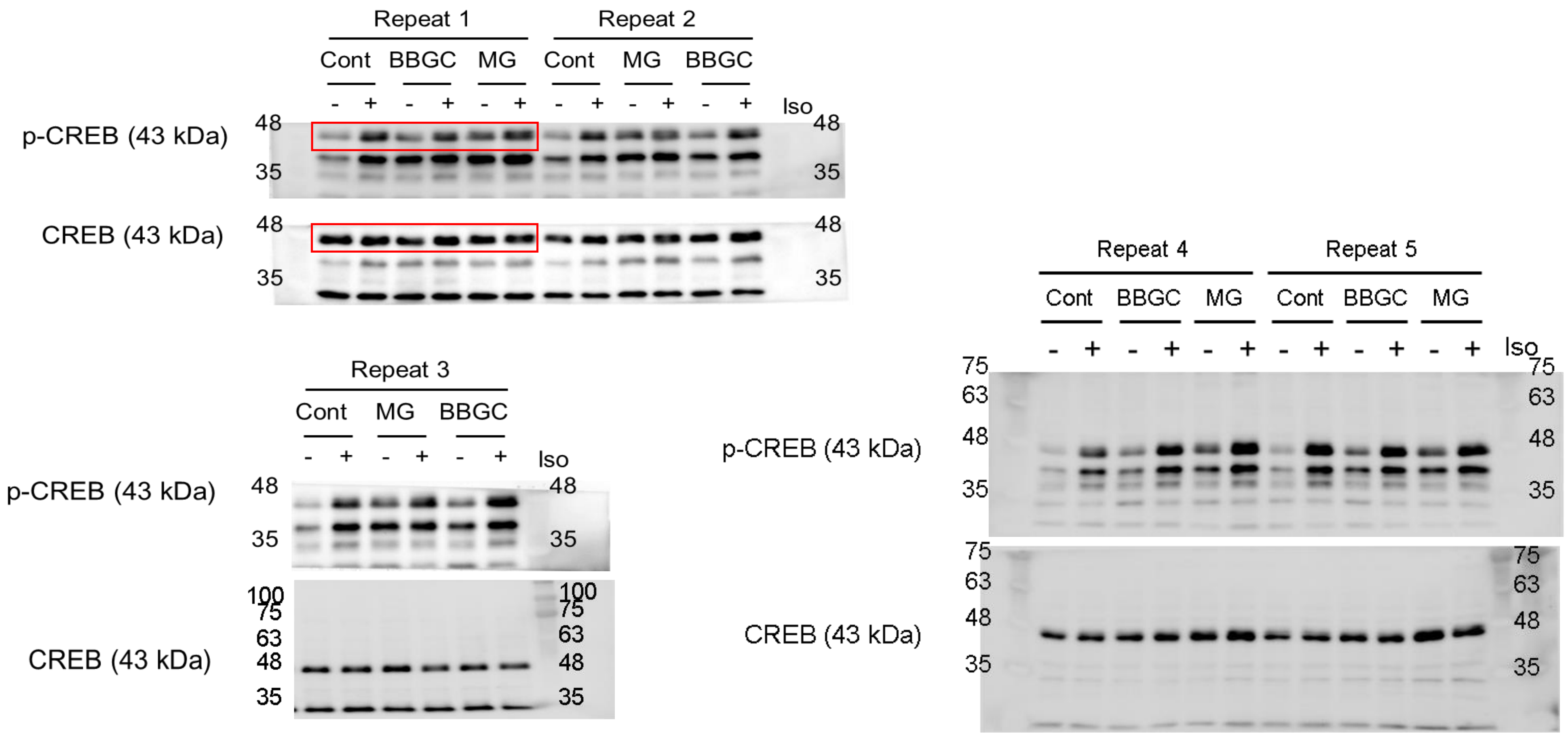

The whole uncropped images of the original western blots in quintuplicate that contributed to the quantitative analysis of p-CREB as shown in Fig. 2(b). The red box indicates the cropped part of the membrane used in Fig 2(a).

Raw Data of Fig. 3(a)

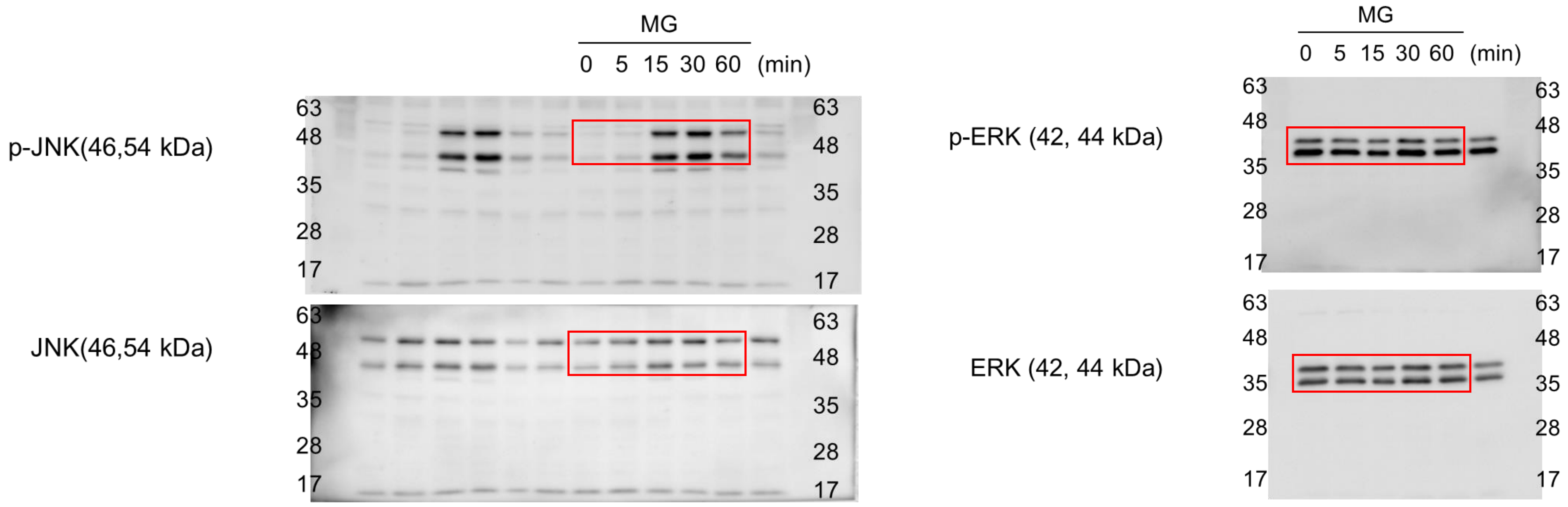

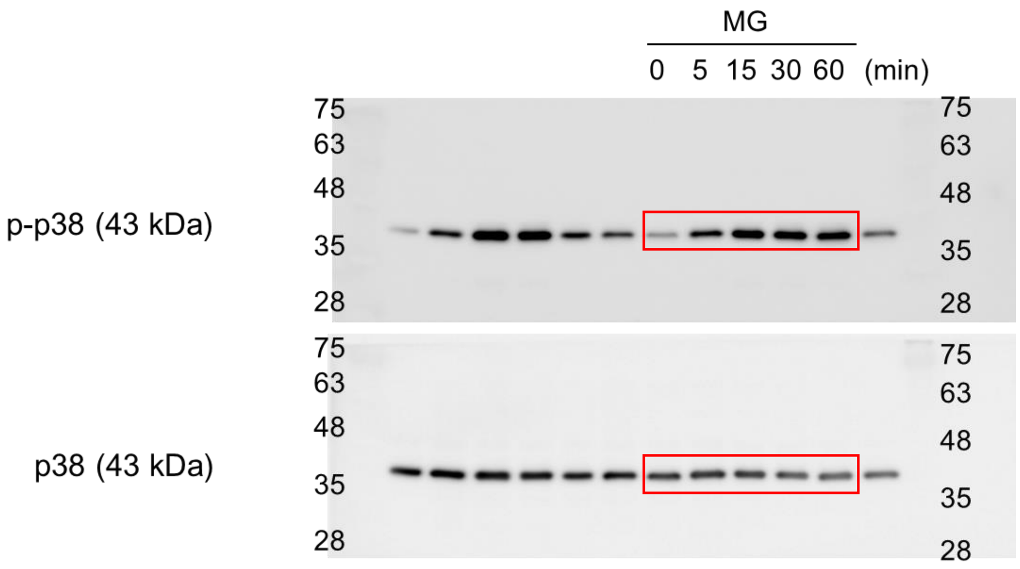

The whole blots of Fig. 3(a). The red box indicates the cropped part of the membrane used in Fig 3(a).

Raw Data of Fig. 3(b)

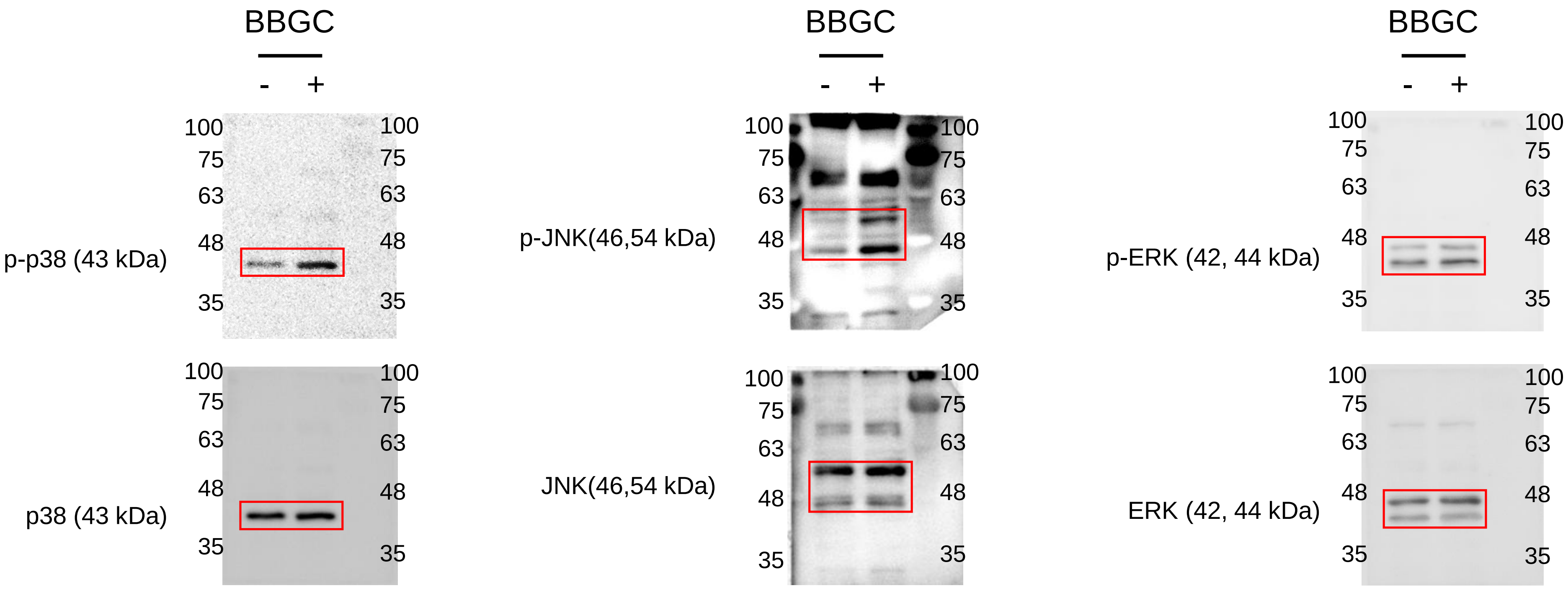

The whole blots of Fig. 3(b). The red box indicates the cropped part of the membrane used in Fig 3(b).

Sextuplicates for Fig. 4(b)

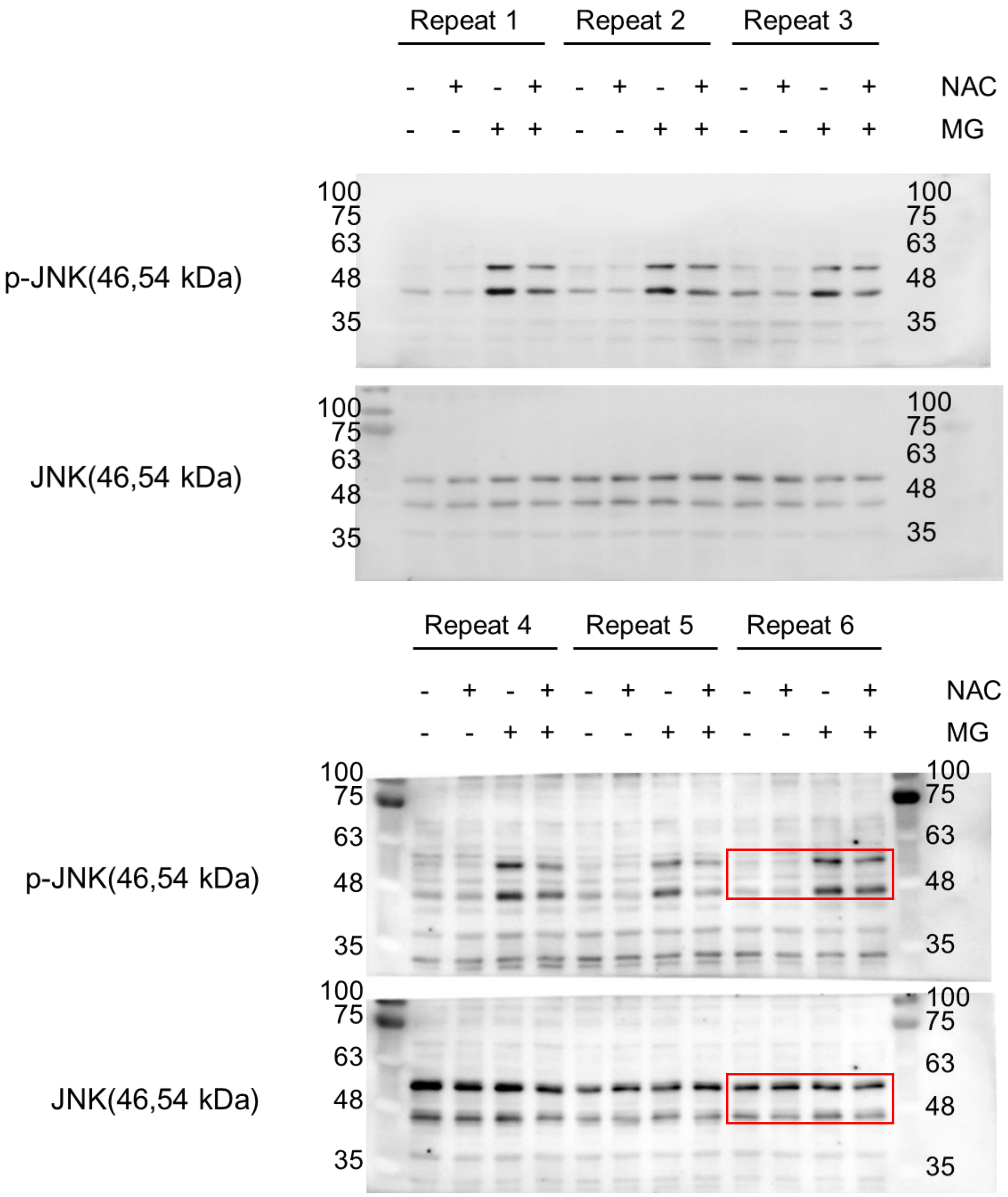

The whole uncropped images of the original western blots in sextuplicate that contributed to the quantitative analysis of p-JNK as shown in Fig. 4(b). The red box indicates the cropped part of the membrane used in Fig 4(a).
